# Noroviruses—The State of the Art, Nearly Fifty Years after Their Initial Discovery

**DOI:** 10.3390/v13081541

**Published:** 2021-08-04

**Authors:** Louisa F. Ludwig-Begall, Axel Mauroy, Etienne Thiry

**Affiliations:** 1 Veterinary Virology and Animal Viral Diseases, Department of Infectious and Parasitic Diseases, Faculty of Veterinary Medicine, FARAH Research Centre, Liège University, 4000 Liège, Belgium; lludwig@uliege.be; 2 Staff Direction for Risk Assessment, Control Policy, Federal Agency for the Safety of the Food Chain, 1000 Brussels, Belgium; axel.mauroy@favv-afsca.be

**Keywords:** norovirus, phylogeny, genome, virion, replication, clinic, epidemiology, detection, immunity, treatment and prophylaxis, evolution, model systems

## Abstract

Human noroviruses are recognised as the major global cause of viral gastroenteritis. Here, we provide an overview of notable advances in norovirus research and provide a short recap of the novel model systems to which much of the recent progress is owed. Significant advances include an updated classification system, the description of alternative virus-like protein morphologies and capsid dynamics, and the further elucidation of the functions and roles of various viral proteins. Important milestones include new insights into cell tropism, host and microbial attachment factors and receptors, interactions with the cellular translational apparatus, and viral egress from cells. Noroviruses have been detected in previously unrecognised hosts and detection itself is facilitated by improved analytical techniques. New potential transmission routes and/or viral reservoirs have been proposed. Recent in vivo and in vitro findings have added to the understanding of host immunity in response to norovirus infection, and vaccine development has progressed to preclinical and even clinical trial testing. Ongoing development of therapeutics includes promising direct-acting small molecules and host-factor drugs.

## 1. Introduction

Human noroviruses (HuNoVs) are recognised as the leading global cause of sporadic and epidemic viral gastroenteritis [1,2] and account for a global economic burden of 60 billion USD, over one million hospitalisations, and 219,000 deaths per annum [2,3]. Despite their importance, the fact that the understanding of HuNoV biology lags behind that of other positive strand RNA viruses, is, in great part, due to the difficulties historically associated with robust in vitro HuNoV propagation. In recent years, however, novel model systems have provided new opportunities for study and previous knowledge gaps are progressively being filled.

Last comprehensively reviewed in 2015 by Robilotti et al., [1], advances in the field in the intervening six years cover nearly all aspects of norovirus biology. Here, we provide an updated overview of norovirus phylogeny, genome organisation, virion morphology, the replicative cycle, and clinical disease. Norovirus evolution, epidemiology, detection and typing, immunity, as well as treatment and prophylaxis are discussed. We highlight notable advances made in each of these subject areas and provide a recap of the novel model systems to which much of the recent progress is owed.

## 2. Phylogeny—Past and Present

**Figure 1:**
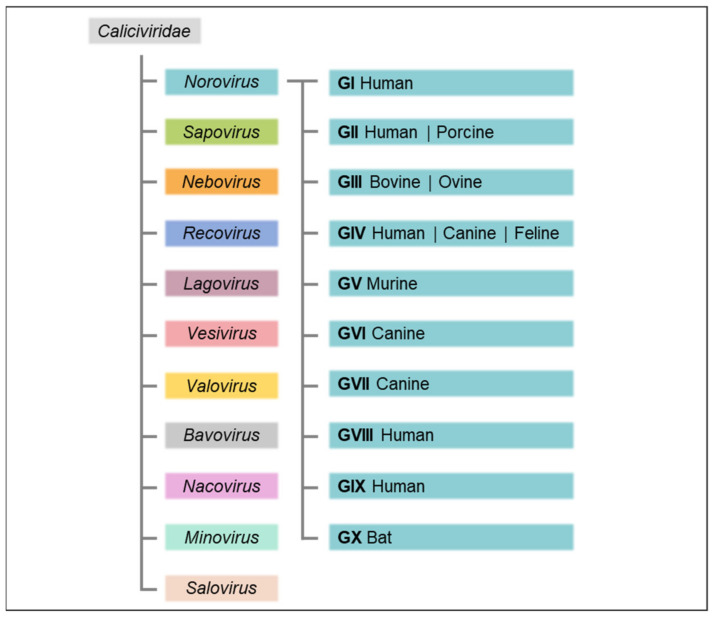
Schematic diagram of the *Norovirus* genus within the *Caliciviridae* family. The ten established genera (GI—GX), as defined by Chhabra et al. 2019, are shown, as well as the species wherein each genogroup has been detected.

The *Caliciviridae* family, small, non-enveloped, positive sense, single-stranded RNA viruses, derives its name from the Latin *calix* for chalice with reference to the cup-shaped depressions that commonly contour the virion surface of caliciviruses. The family is comprised of eleven approved genera: *Norovirus*, *Sapovirus*, *Nebovirus*, *Recovirus*, *Lagovirus*, *Vesivirus*, *Valovirus*, *Bavovirus*, *Nacovirus*, *Minovirus*, and *Salovirus* (Figure 1), which are distinguished based on over 60% amino acid sequence difference in the complete major capsid protein (VP1) sequence [3] and infect a wide range of host species causing a variety of mainly species-specific diseases [4].

The genetically diverse noroviruses, which infect a broad range of mammalian hosts, derived their name from the city of Norwalk, Ohio, where an acute gastroenteritis outbreak was caused by the prototypic Norwalk virus in 1968 [5]. In the early 2000s, classification into norovirus genogroups and genotypes was initially based on amino acid sequence analysis of the complete VP1 capsid protein, with an amino acid divergence of 14.1% within a genotype and an adjusted cut-off threshold of a minimum of 15% pairwise difference proposed for classification of new genotypes [6,7].

In 2013, the international *Norovirus Classification Working Group* put forth a proposal for a unified norovirus nomenclature and genotyping, whereby noroviruses were genetically classified into six established genogroups (GI–GVI), with a seventh proposed (GVII). Genogroups were further divided into at least 38 genotypes based on phylogenetic clustering of complete VP1 amino acid sequences [8,9]. The GII.4 strains were additionally subtyped into variants based both on phylogenetic clustering and on the condition of their having become epidemic in at least two separate geographical locations and were named according to year and location of the first full-length capsid sequence in the public domain. To account for the common occurrence of recombination in the overlapping region between the first two of three open reading frames (ORF1/2) encoded by noroviruses, a dual nomenclature system based on complete capsid sequences in ORF2 and partial sequences of the RNA-dependent RNA polymerase (RdRp) region in ORF1 was established [8]. Naming of “orphan” ORF1 polymerase types, also known as “obligatory norovirus recombinants” and designating known RdRp sequences lacking attributed capsid sequences but promiscuously associated with capsids of different genotypes, followed a preliminary alphabetical naming system [8].

Adhering to the established criteria for genotype attribution and numbering of complete capsid sequences, the prior classification was recently updated to encompass ten accepted genogroups (GI to GX) and 49 confirmed genotypes (Figure 1), as well as two tentative genogroups (GNA1 and GNA2) and three proposed genotypes [10]. To more easily accommodate ORF1/2 recombination of noroviruses and to eliminate the necessity of the letter-based orphan ORF1 naming system, partial RdRp sequence clusters were grouped into two tentative polymerase (P)-groups as well as 60 accepted and 14 tentative P-types independently of the classification of their capsid genogroups or genotypes. Accordingly, nine VP1 genotypes in GI, 26 in GII, three in GIII, two each in GIV, GV, and GVI, and one each in GVII to GX are currently recognised; of the P-types, 14 cluster in GI, 37 in GII, two in GIII, one in GIV, two each in GV and GVI, and one each in GVII and GX. The previous dual typing nomenclature of norovirus strains was abandoned in favour of an updated version first listing the capsid genotype followed by the P-type between brackets (e.g., previous designation GII.P12-GII.3 and current designation GII.3[P12]). For strains where ORF2 and ORF1 amino acid sequences cluster in different genogroups, the designations are Genogroup.genotype[Pgroup.P-type] (e.g., previous designation GVI.P1-GIV.2 and current designation GIV.2[GVI.P1] [10].

Genogroups GI, GII, GIV, GVIII and GIX (previously GII.15) infect humans and cause acute gastroenteritis [10,11]. Other species in which noroviruses have been detected include pigs (GII) [12], cattle and sheep (GIII) [13,14,15], rats and mice (GV) [16], dogs (GVI) [17], and bats (GX) [18]. Tentative new genogroups GNA1 and GNA2 are detected in harbour porpoises [19] and sea lions [20], respectively.

## 3. Genome Organisation

The linear, positive sense, single-stranded RNA genomes of noroviruses are between 7.3 and 7.5 kilobases (kb) in length [21]. A subgenomic RNA identical to approximately the last 2.3 kb of the genome is found in viral particles and is expressed, at higher levels than the viral genomic RNA, in infected cells [22]. The 5′ ends of norovirus genomic and subgenomic RNA are linked to viral protein VPg [23,24,25], the 3′ ends are polyadenylated [26]. At their extremities, norovirus genomes contain short untranslated regions (UTRs) [27] which contain evolutionarily conserved RNA secondary structures that extend into the coding regions and are repeated throughout the genome, playing functional roles for viral replication, translation, and pathogenesis by binding viral and host factors [28]; a highly conserved non-coding RNA stem-loop structure upstream of the start site for subgenomic RNA initiation at the overlap of ORFs 1 and 2 has been identified as the core promoter for norovirus subgenomic RNA synthesis by binding with the viral RNA-dependent RNA polymerase (RdRp) [21,29,30,31].

**Figure 2:**
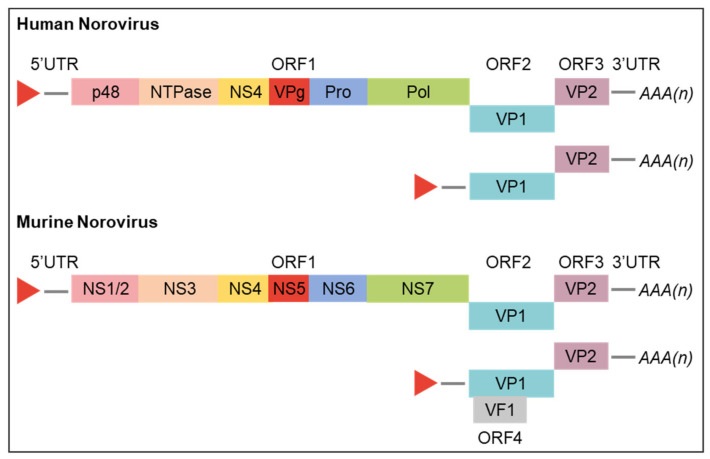
Schematic diagram showing the organisation of norovirus genomes. (**Above**) The human norovirus genome is covalently attached to genome-linked viral protein VPg at the 5′ end and is polyadenlyated at the 3′ end. The genome is divided into three open reading frames (ORFs). ORF1 is translated as a polyprotein, which is cleaved by the viral protease (Pro) to produce the nonstructural proteins (p48, NTPase, p22, VPg, Pro, and Pol). ORF2 and ORF3 are translated from a subgenomic RNA. They encode the major structural protein, VP1, and the minor structural protein, VP2, respectively. The 5′ and 3′ genome extremities contain short untranslated regions (UTRs). (**Below**) The murine norovirus shares a similar genome organisation but has an additional fourth ORF, which overlaps with ORF2 and is also translated primarily from subgenomic RNA into the virulence factor 1 (VF1) protein; adapted from Thorne and Goodfellow 2014 [21].

The norovirus genome is organised into three or, for MNV, four ORFs (Figure 2) [32]. The 5′ proximal ORF1 encodes a large polyprotein that is co- and post- translationally cleaved by a virus-encoded protease into six nonstructural proteins (NS) involved in replication complex formation (NS1/2, NS3, and NS4), genome linkage (NS5 and VPg), polyprotein processing (NS6), and genome replication (NS7 and RdRp) [21,33,34]. ORF2 and ORF3, both translated from subgenomic RNA, encode the structural components of the virion, major viral protein (VP1) and minor viral protein (VP2), respectively. ORF4, unique to MNVs, overlaps ORF2 and is also translated primarily from subgenomic RNA; it encodes the virulence factor 1 (VF1) which is involved in regulation of innate immunity and apoptosis [32,35]. The functions of various norovirus proteins are discussed further below in the context of the norovirus replicative cycle and were recently reviewed by Campillay-Véliz et al. [36].

## 4. Virion Morphology

Typically, a norovirus capsid is 27–30 nm in diameter and exhibits T = 3 icosahedral symmetry, with calicivirus, characteristic cup-like depressions localised at the three- and five-fold symmetry axes. Each capsid is composed of 180 copies of monomeric major structural protein VP1 which form 90 dimeric capsomers [37,38]. Each VP1 comprises a short N-terminal arm of unknown function, a shell domain (S), and a protruding domain (P) [38]. The well-conserved N-terminal S domain faces the interior of the capsid and forms a continuous surface surrounding the viral RNA. The P domain, linked to the S domain through a flexible hinge, corresponds to the C-terminal part of VP1. It is postulated to confer increased stability to the icosahedral capsid and to provide a control for the size of viral particles [39]. The P domain is further divided into a proximal P1 stalk subdomain at the base of the arches and the highly variable distal P2 subdomain. Localised at the tips of the arches, the exposed P2 subdomain interacts with neutralising antibodies and contains the defined host receptor binding site for MNVs [40] and putative receptor binding site for HuNoVs [41,42,43,44]. Cryo-electron microscopy has revealed distinct P domain conformations for both MNV and certain HuNoV strains (GII.10) that are dependent on buffer pH and the presence of bile salts. A “floating” P domain is described to hover above the shell in physiological buffers but dynamically rotates and contracts onto the shell surface in the presence of bile salts in a probable mechanism that enhances P domain stabilisation and receptor affinity [45]. The unique flexibility of the *Caliciviridae* P domain in response to environmental cues is described in the 2019 review by Smith and Smith [46], the role of bile salts and their interactions with viral capsids (also further discussed below) were recently reviewed by Tenge et al., 2021 [47]. Interestingly heterologous expression of larger GII.4 variant VLPs exhibiting T = 4 icosahedral symmetry (T = 4 particles assembled from 240 copies of VP1) was recently described [48].

Minor structural protein VP2 [49], encoded by all caliciviruses, is located at the interior of the viral capsid and bound to a conserved motif in the VP1 S domain. It is postulated to be involved in MNV encapsidation via an interaction with viral genomic RNA [21,50] and acidic regions of VP1 [33] and is held to regulate expression and stability of VP1 in HuNoVs [27]. VP2 integrity has been shown to be essential for productive replication of infectious feline calicivirus (FCV) [51]. Feline calicivirus VP2 forms a portal-like assembly following host cell receptor engagement and has been hypothesised to function as a channel for viral genome release from the endosome into the host cell cytoplasm [52]; morphological changes upon viral entry may be a general phenomenon shared amongst all calicivirus VP2s.

## 5. Replicative Cycle

### 5.1. Attachment, Receptor Engagement, Endocytosis, and Uncoating

As the initial step of the norovirus replicative cycle and decisive early determinant of cell tropism, host range, and pathogenesis, the multi-phasic process of viral entry commences via virion attachment to the cell surface [53]. Attachment of noroviruses is mediated by binding of the virus to both cell-associated and soluble host factors [44].

HuNoVs are bound by histo-blood group antigens (HBGAs), as evidenced by in vitro assays [54] as well as multiple volunteer studies documenting the correlation between long-term resistance to infection with certain HuNoV strains and FUT2 gene-mediated genetic polymorphisms that determine host secretor status [55,56]. The ability to secrete a diverse set of fucosylated HBGAs into body fluids and to express HGBAs on mucosal cells (secretor) is associated with HuNoV susceptibility. Expressing only a limited array of HBGAs (non-secretors) is linked to resistance to certain HuNoV strains (including genogroups GI.1 and prevalent GII.4) [57,58]. While non-secretors experience infections with a lesser variety of norovirus strains, their resistance to HuNoV has recently been shown not to be absolute and they can become infected by secretor-independent strains (GII.3, GII.7, and GII.6), newly implicating non-HGBA ligands (fucosylated and sialylated carbohydrates [59]) and co-factors in HuNoV binding [44,60,61]. While canine (GVI) and bat (GX) noroviruses attach to HBGAs similar to HuNoVs [62,63], bovine GIII.2 strains do not bind the same carbohydrate moieties as HuNoVs but recognise α-Gal 1,3 motif [64,65,66]. For MNVs, non-essential carbohydrate attachment factors including heparan sulfate proteoglycans and terminal sialic acid have been shown to enhance viral VP1 binding in a strain-dependent manner [43,67,68]. Notably, sialic acids have also been implicated in facilitating the attachment of bovine noroviruses and FCV to susceptible cells [69,70].

Suggested host and microbial cofactors that enhance norovirus attachment to cells (also in a strain-dependent manner) include bile acids (MNV and HuNoV) [47,71,72,73], phospholipids (MNV) [74], and divalent cations (MNV) [71].

The second step of viral entry is the engagement of host receptors to actively promote viral access to cells. CD300lf, an immunoglobulin domain-containing integral membrane protein expressed in myeloid cells, lymphoid cells, and intestinal epithelial tuft cells [75], has been identified as the primary physiologic cellular MNV receptor [43]. It functions by binding the apical side of the P2 subdomain and is essential for infection of diverse MNV strains both in vitro and in vivo, independent of infection route [40]. Its paralogue CD300ld has also been demonstrated to be sufficient for MNV infection in vitro. Ectopic expression of murine CD300lf on human and other mammalian cells has been shown to be sufficient to confer cross-species susceptibility, effectually breaking the species barrier and allowing MNV replication in non-murine cells [43]. Human CD300lf is not a receptor for HuNoVs and the HuNoV receptor remains unknown [40].

The details of which mechanisms are involved in the endocytic internalisation of HuNoV particles following receptor engagement are unknown. For MNVs, entry into permissive macrophages and dendritic cells is known to be rapid, requiring host cholesterol and dynamin [76,77]. This viral endocytosis is independent of pH [78], clathrin, and caveolae, and is neither mediated by phagocytosis nor micropinocytosis [76]. For bovine noroviruses, VLP internalisation into permissive cells involves both the cholesterol-dependent pathway and macropinocytosis [69]. Norovirus attachment and entry were most recently reviewed in the 2019 publication by Graziano et al. [44]; norovirus, host, and microbiota interactions were outlined by Walker and Baldridge the same year [79].

After endocytosis, endosomal escape and viral uncoating are required to release the viral genome into the host cytoplasm. While the process remains unsolved for noroviruses, a recent near-atomic resolution analysis of FCV yielded important basic information regarding these important last steps of calicivirus entry. In the process of clathrin- and pH-dependent endocytosis, binding of FCV to its receptor feline junctional adhesion molecule A was shown to induce formation of a portal-like assembly made up of twelve copies of VP2 arranged with their hydrophobic N termini pointing away from the virion surface around a pore in the capsid shell. The funnel-like structure is hypothesised to function as a channel for the delivery of the viral genome through the endosomal membrane into the cytoplasm of a host cell, thereby initiating infection [52].

### 5.2. Translation and Polyprotein Processing

Following its release into the cytoplasm of a permissive cell, the VPg-linked norovirus RNA acts as a messenger RNA (mRNA) template for an initial round of viral RNA translation. Attached covalently to the 5′ end of the genome, norovirus VPg (NS5) functions as a cap substitute to recruit eukaryotic initiation factors and mediate translation of viral RNA into protein via multiple direct interactions with the cellular translational apparatus of the host cell and core stress granule components [23,80,81,82,83,84]. Interactions between various host cell RNA-binding proteins and conserved RNA secondary structures of complementary sequences at the 3′ and 5′ genome extremities are further postulated to enhance and regulate viral protein translation, putatively by stabilising sequence-mediated, long-range physical RNA interactions that promote genome circularisation [28,85].

Translation of the viral proteins VP1 and VP2 is regulated by the 3’ terminus of the polycistronic subgenomic RNA [27]. VP1 and VP2 translation occurs primarily from the ORFs of the subgenomic RNA generated via premature termination and/or internal initiation during RNA synthesis (see below). Following its transcription from genomic RNA by the norovirus nonstructural proteins, subgenomic RNA is expressed at higher levels than the viral genomic RNA in infected cells [22], in a probable strategy to augment levels of VP1 production for virus assembly [21]. Translation of ORF4 in MNV from subgenomic RNA yields VF1 which has been implicated in interfering with innate immune signalling at the cellular level and was recently found to delay the upregulation of IFN-β and other Interferon stimulated genes (ISGs) in vitro [32].

### 5.3. Viral Genome Replication

Once translated, the ORF1 polyprotein is co- and post-translationally cleaved by the viral protease (NS6) to release NS precursors and mature viral proteins (NS1/2 to NS7) [34,36,86], which then serve to assemble the replication complex by recruitment of cellular membranes to the perinuclear region of the cell [87].

Murine norovirus NS1/2, the least conserved norovirus NS [33], is hypothesised to be one of the main drivers of replication complex formation by associating with components of the endocytic and secretory pathway together with co-localising NS4 [88,89]. NS1/2 contains an N-terminal disordered region and a C-terminal predicted transmembrane domain [90]. Murine norovirus NS1/2 has been shown to induce rearrangement of the endoplasmic reticulum. It is implicated in viral persistence in vivo [91] and, once unconventionally secreted via caspase-3 cleavage, is essential for intestinal pathogenesis of MNV infection and resistance to endogenous IFN-γ [92]. Its HuNoV equivalent (p48) promotes Golgi disassembly dependent upon the C-terminal hydrophobic region and disrupts expression and trafficking of cell surface proteins by interfering with cellular vesicle transport [93,94]. A secreted form of HuNoV NS1 is also observed [92].

As a constituent of the MNV replication complex, NS4 localises to endosomes [87,88]. HuNoV NS4 (P22) has been shown to induce Golgi disassembly [95] and has been identified as a key determinant in the formation of membrane alterations by HuNoVs [94]. MNV and HuNoV NS4 both inhibit cellular protein secretion (mildly in MNV and potently in HuNoV) [88,95].

While NS1/2 and NS4 are acknowledged to be key main mediators of replication complex formation, NS3, to which RNA chaperon and helicase activities have been attributed [96,97], has also been shown to localise to cellular membranes [87]. HuNoV and MNV NS3 both induce formation of motile membrane-derived vesicular structures that colocalise with the Golgi apparatus and the endoplasmic reticulum [94,98].

Norovirus genome replication occurs via a negative-strand intermediate [21]; subsequent to the initial round of translation of the incoming parental positive-strand RNA, this mRNA serves as a template for the synthesis of negative-strand RNA from its 3′ end and the formation of a double-stranded replicative form. Then, the negative-sense genomic and subgenomic RNAs are used as templates for the synthesis of positive sense genomic and subgenomic RNAs [21]. These transcription reactions are catalysed by the RNA-dependent RNA-polymerase (RdRp, NS7), using de novo mechanisms for synthesis of negative-stranded RNA [99,100], and VPg-dependent mechanisms of positive sense genomic and subgenomic RNA synthesis in which the NS7 uses multifunctional VPg as a proteinaceous primer [23,25,101]. Multifunctional VPg [24] plays various roles in norovirus replication including induction of higher-order RdRp multimer or tubular fibril formation and enhancement of MNV RdRp activity [25]. Furthermore, induction of a G0/G1 cell cycle arrest which provides a favourable environment for viral replication (the cellular growth phase provides high levels of ribonucleotides and a high translation efficiency) has been demonstrated to be a conserved function of norovirus VPgs [101]. Two, not mutually exclusive, models have been proposed for the generation of norovirus subgenomic RNA. On the basis of the detection of negative-sense subgenomic RNA copies in Norwalk virus replicon-bearing and MNV infected cells [29,102], the premature termination model proposes synthesis of negative-sense subgenomic RNA linked to an unidentified termination signal, and subsequent generation of positive sense subgenomic RNA from this template. The internal initiation model postulates that the highly conserved stem-loop structure upstream of the subgenomic start site in the negative-sense genomic RNA acts as the core of an internal subgenomic promoter and binds to the RdRp to direct initiation at the overlap of ORFs 1 and 2. In this case, newly synthesised subgenomic RNA may function as a template for further rounds of replication via a negative-sense subgenomic RNA intermediate [28,29,30,31].

### 5.4. Assembly and Exit

Self-assembly of VP1 into VLPs that are morphologically and antigenically comparable to native virions [39] suggests that VP1 alone may be able to drive assembly of infectious norovirus particles. While not essential for assembly, the 3′ UTR of the Norwalk mRNA can stimulate VP1 expression via putative RNA-capsid interactions and the presence of VP2 is held to enhance the stability of nascent particles [27,103,104]. Associated with a conserved acidic motif in the VP1 S domain at the capsid interior [33,50], the highly basic VP2 may provide the link between capsid subunits and acidic viral RNA [21,50].

Upon completed assembly, virion exit is the last step of the replicative cycle. Active viral replication of MNVs in permissive cells, and indeed the expression of the MNV polyprotein alone, has been shown to regulate and induce apoptosis and programmed cell death in conjunction with downregulation of prosurvival factor survivin in infected cells [105,106]. While its role in viral exit remains undetermined, inhibition of apoptosis has been shown to accelerate cell death, change the death pathway to rapid necrosis, and to ultimately result in an over 10-fold reduction in infectious norovirus yield [107].

**Figure 3:**
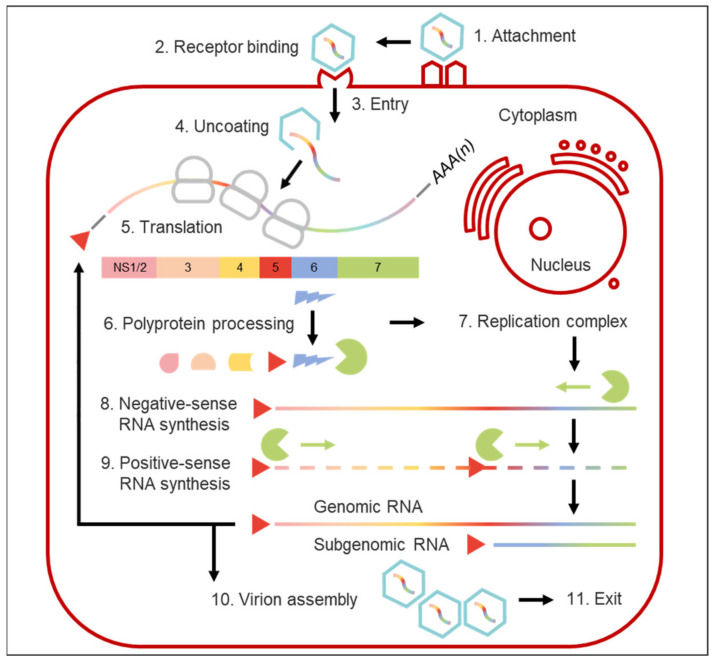
Outline of the norovirus replication cycle. Human and murine noroviruses (turquoise hexagons) attach to the cell surface using carbohydrate attachment factors and cofactors (**1**). To mediate entry, binding to a protein receptor is required (**2**). After entry (**3**) and uncoating (**4**), the incoming viral genome is translated through interactions with genome-linked protein VPg (nonstructural protein NS5, red triangle) at the 5′ end of the genome and the cellular translation machinery (**5**). The open-reading frame 1 polyprotein is co- and post-translationally cleaved by the viral protease (NS6, blue flash) (**6**). The replication complex is formed by recruitment of cellular membranes to the perinuclear region of the cell, through interactions with NS1/2 (rose shape) and NS4 (yellow shape) (**7**). Genome replication occurs via a negative-strand intermediate (dashed line) (**8**), and genomic and subgenomic RNA (unbroken lines) are generated by the viral RNA-dependent RNA polymerase (NS7, green jagged circle), using de novo and VPg- or internal promoter-dependent mechanisms (**9**). Replicated genomes are translated or packaged into the capsid (in the case of whole-length genomes), composed mainly of viral protein 1 (VP1), for virion assembly (**10**) and exit (**11**).

While MNVs lytically infect innate immune cells including macrophages and dendritic cells in vitro [16], the nature of in vitro B cell infection by HuNoVs and MNVs is distinct, since mature B cells are infected non-cytopathically [108,109], suggesting that different mechanisms of cellular regulation and cell exit can be employed by noroviruses. The paradigm that nonenveloped viruses must lyse their target cells in order for progeny virions to be released extracellularly has further been challenged by the discovery that, amongst other enteric viruses, noroviruses can be secreted from cultured cells inside extracellular membrane-bound vesicles and that they are shed in faeces within vesicles of exosomal or plasma membrane origin presenting highly virulent units of faecal-oral transmission [110]. An outline of the entire norovirus replication cycle is provided in Figure 3.

## 6. Clinical Aspects of Norovirus Infection

### 6.1. Human Noroviruses

Human noroviruses cause both sporadic and epidemic viral gastroenteritis [1,111]. After a short incubation period of 24–48 h [112], clinical symptoms typically last for two to three days [1], followed by a median of four weeks of post-clinical shedding [113] with peak viral titres varying between 10^5^ and 10^9^ genome copies/g of faeces [114]. Characteristic symptoms of HuNoV infection are acute onset of watery, non-bloody diarrhoea, and projectile vomiting [115]. Other symptoms include abdominal cramps, nausea, bloating, mild fever, chills, headaches, and myalgia [116,117,118]. While self-limiting gastrointestinal infections are the norm, more severe intestinal pathologies such as necrotising enterocolitis in neonates [119,120], post-infectious irritable bowel syndrome [121], and exacerbation of inflammatory bowel disease [122] have been described. Atypical extraintestinal pathologies such as seizures in young children [123,124,125], encephalopathy [126], and acute liver dysfunction [127,128] have also been reported in association with norovirus infections. Norovirus RNA has been detected in sera [129] and cerebrospinal fluids [126] of infected individuals, suggesting possible spread to peripheral tissues.

Despite typically eliciting severe gastroenteritis, HuNoVs cause only modest intestinal pathologies. Histopathological changes in the small intestine include broadening and shortening of the microvilli, crypt hypertrophy, as well as increased epithelial mitoses and apoptosis [130]. Decreased brush border enzyme activity; transient malabsorption of D-xylose, fat, and lactose; disruption of epithelial barrier functions; reduction of tight junctional sealing proteins; and stimulation of active anion secretion suggest that both a leak flux and alterations of secretory and/or absorptive processes cause HuNoV-induced diarrhoea [131,132,133]. Vomiting episodes may be linked to abnormal gastric motor functions and delays in gastric emptying, however, the underlying pathophysiology remains unclear [134]. Asymptomatic infections and viral shedding similar to that of symptomatic infections [114] have been both experimentally observed in volunteer studies [135] and detected in various epidemiological analyses of clinically healthy individuals and those with various underlying illnesses resulting in impaired immunity [136,137,138,139]. Customarily, an acute and self-limiting illness, HuNoV infection can become persistent in the elderly [140], malnourished, and/or immunocompromised (individuals with genetic or acquired immune deficiencies, cancer patients undergoing treatment, transplant patients) [141,142,143,144,145]. These individuals often experience severe, even lethal, persistent or recurring NoV infections during which prolonged diarrhoea and vomiting can lead to weight loss and malabsorption; in these patient cohorts, viral RNA remains detectable in stool samples for months to years [141,146,147,148].

### 6.2. Animal Noroviruses

Animal noroviruses have been linked to gastroenteritis outbreaks and acute diarrhoeic episodes of varying severity in cattle [13], pigs [149,150,151], cats (including a captive lion cub) [152,153], and dogs [154]. While a clinical association typically exists in these domesticated mammalian hosts, asymptomatic infections have been observed (at lower prevalences) in epidemiological screening studies [15,66,155]. The only documented GIII sheep noroviruses were reportedly detected in animals that showed no obvious clinical signs [156]. In wild animals such as bats [18], harbour porpoises [19], and Californian sea lions [20], where noroviruses are typically detected in the context of metagenomics analyses and/or retrospective analyses of stored samples, a potential disease association often remains undetermined.

### 6.3. Murine Noroviruses

Murine noroviruses have been isolated from asymptomatic wild populations of both field and wood mice (*Apodemus agrarius* and *Apodemus sylvaticus*) [157,158] and have been detected in various cohorts of domesticated mice (*Mus musculus*), including mice sold as pets or snake food, show animals, and those bred for academic research [158].

Indeed, MNVs are recognised as one of the most prevalent, albeit often undetected, pathogens of contemporary laboratory mice, as evidenced by serologic testing and reverse transcription polymerase chain reaction (RT PCR) screening [159,160,161]. Thirty fully sequenced MNV strains have been isolated from specific-pathogen-free mice in academic research colonies across the globe; while these strains comprise a single genetic cluster, they broadly segregate into two categories regarding their pathogenesis and disease profile [162]. The prototype acute strain MNV-1, which infects immune cells in the gut-associated lymphoid tissue, reaches peak intestinal titres 1–2 days post-infection (dpi) and is cleared by 7–14 dpi. Persistent strains MNV-3 and MNV-CR6 can establish life-long infections, linked to replication in the caecum, colon, mesenteric lymph nodes, and rare intestinal epithelial tuft cells [161,163,164]. Persistent asymptomatic infection with typically nonpersistent strain MNV-1 CW3 has been associated with adaptive changes to viral proteins NS1/2, NS7, and VP2 [91,165].

Notwithstanding differences in clearance kinetics and cell tropism, all MNV strains elicit subclinical infections of the intestine, lacking any association with diarrhoea or other overt disease in wild-type juvenile and adult mice, and certain knockout, strains. A MNV-1-induced decrease in faecal consistency as measured by visual scoring of faecal samples of immunocompetent mice remains the only modest disease association (MNV-3 failed to induce this pathology) [162]. Despite a subclinical presentation, quantifiable intestinal pathology and detection of viral RNA in the liver, spleen, mesenteric lymph nodes, and proximal intestine (but not in the lung, brain, blood, or faeces) have been described in association with experimental MNV infection of wild-type hosts [16,160,166,167].

In severely immunodeficient adult mice lacking functional components of the innate immune system and interferon pathways, MNV infection has been shown to be associated with lethal disease [16,167]. Following oral MNV-1 inoculation, mice deficient in signal transducer and activator of transcription 1 (STAT1) and recombination-activating gene 2 (RAG2) rapidly succumb to systemic disease associated with severe weight loss, diarrhoea, bloating, pathologies in intestinal and peripheral tissues, and the presence of viral RNA in all organs. Persistent strains MNV-3 and MNV-CR6 cause less overt symptoms than MNV-1 in interferon-deficient mice [168].

Recently, self-resolving diarrhoea in the absence of systemic disease was reported in MNV-infected wild-type neonatal mice, mirroring key clinical features of HuNoV disease; diarrhoeic episodes were neither associated with disruption of the intestinal epithelium nor notable inflammation. Oral MNV-1 inoculation, and to a lesser extent that of MNV-3 and MNV-CR6, caused acute diarrhoea in three-day-old BALB/c mice [169].

## 7. Human Norovirus Epidemiology and Transmission

### 7.1. The Societal Burden of Norovirus Infections and the Role of Genotype GII.4

As major aetiologic agents of global sporadic and epidemic non-bacterial gastroenteritis [1,111], HuNoVs cause significant morbidity and mortality in developing countries and engender enormous economic losses in developed countries [170]. Causing a median number of 669 million illnesses and an estimated 219,000 deaths across all ages per year globally, HuNoVs have been calculated to result in a yearly total of 4.2 billion USD in direct health care costs (outpatient visits and hospitalisation) and 60.3 billion USD in societal costs (productivity losses due to absenteeism or mortality) [170].

GII.4 infections, which are responsible for the majority of past HuNoV outbreaks (55–85%) and also sporadic cases, have been associated with a higher probability of severe outcomes and lead to higher hospitalisation and mortality rates [171]. GII.4 noroviruses have been the predominant genotype circulating in humans for over two decades. Until 2012, novel circulating GII.4 strains emerged every two to three years and replaced their predecessors in an immune-driven selection process [172,173,174]. Since 2012, the same capsid type, GII.4 Sydney, has been the dominant genotype [175,176]. Postulated mediators for the GII.4 dominance include selective advantages and improved adaptation to host receptors via physicochemical P2 changes in the virion of new GII.4 subtypes and the evasion of herd immunity against predominant genotypes [177,178,179,180]. The elevated number of novel nonsynonymous mutations in GII.4 capsid sequences and changing HBGA binding patterns [181], as well as intragenotypic recombination have long been postulated to be a driving force of GII.4 noroviruses. Strain-dependent differences in norovirus molecular evolution via the accumulation point mutations are briefly discussed below. Complex patterns of recombination within the GII.4 lineage were recently discussed in our 2018 review on norovirus recombination [182]. The position of the rapidly evolving dominant GII.4 variants has only recently been challenged by emergence and re-emergence of different intra- and intergenotype recombinants modifying long-term global norovirus genetic diversity trends [11,180,183,184,185,186,187].

### 7.2. Norovirus Shedding and Human Infectious Dose

Norovirus particles are shed by healthy individuals for a median of seven days [113]. In patients with waning immunity, long shedding can occur for weeks to months (in rare cases even years) via the faeces or vomit of both infected symptomatic and asymptomatic patients [139,188,189]. Almost half of all symptomatic individuals in long-term care facilities were recently shown to shed norovirus for at least 21 days [190], while a recent cohort study of immunocompromised individuals identified a proportion of chronic shedders (16.4%) that shed infectious virus for 37 to > 418 days [189]. While the main norovirus transmission route is faecal-oral transmission, with faecal loads reaching up to 10^9^ genomic copies/g faeces [113,114], transmission via vomiting has also been identified as a risk [191]. Unlike shedding through stool, vomiting is more likely to result in significant environmental contamination, leading to transmission through fomites and airborne vomitus droplets (1.7 × 10^8^ genome equivalent copies are typically shed in emesis (circa 4 x 10^4^ genomic equivalent copies/mL vomitus) [192,193,194]. The high doses of virus shedding stand in clear contrast to the low 50% human infectious dose which has been calculated to lie between 1320 and 2800 genome equivalents [194].

### 7.3. Transmission Routes

**Figure 4:**
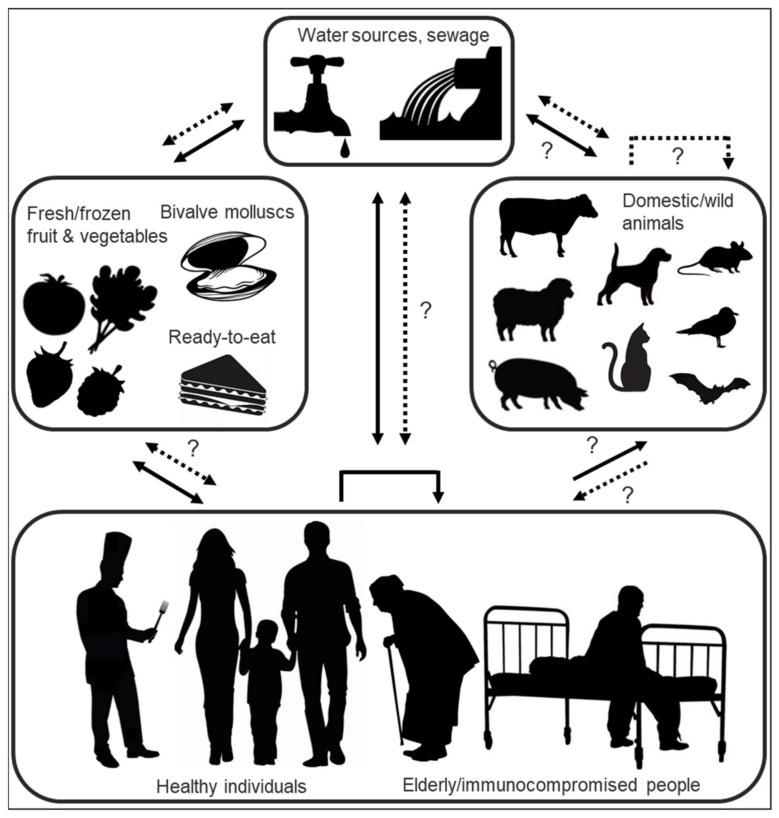
Norovirus transmission. Transmission routes of human noroviruses (solid arrows) and animal noroviruses (dashed arrows) are shown. Unconfirmed transmission is indicated by a question mark. The dashed arrow indicating transmission between domestic/wild animals denotes species-specific transmission; the question mark accompanying it indicates putative interspecies transmission. Adapted from Mathijs et al., 2012 [220] and Ludwig-Begall et al., 2018 [182].

Highly tenacious and resistant in the face of various virucidal treatments and decontamination methods [195,196,197], HuNoVs are transmitted either via direct person-to-person contact or by consumption of contaminated water or food (Figure 4) [198]. Infectious viruses can enter environmental waters either via direct discharge or release of improperly treated sewage from industrial-scale or small private wastewater treatment plants, discharges from vessels, as well as urban runoff, the latter especially in times of flooding or heavy rainfall which have been linked to a high prevalence of HuNoVs in coastal waters [199,200,201,202]. Suspended or precipitated noroviruses have been shown to retain infectivity for weeks to months [200,203,204,205] and have been detected up to 10 km distant from their discharge point [202,206].

The foodborne proportion of HuNoV outbreaks is estimated at 14% [198]. Foods implicated in outbreaks are contaminated either directly with faecal matter at the source or by infectious food handlers [207]. The most common food vehicles remain fresh or frozen soft fruits and vegetables, ready-to-eat foods (such as sandwiches and salads [208,209,210]) which require handling but no or little subsequent cooking, and undercooked or raw seafood (bivalve molluscs) [211]. Bivalve molluscs, including cockles, mussels, clams, scallops, and oysters, accumulate noroviruses via filter feeding; depuration practices, which aim at eliminating such bioaccumulated pathogen charges, are unsuccessful in the face of norovirus contamination. Increasingly, this effect is attributed to the fact that noroviruses are not only filtered and concentrated through nonspecific interactions, but are also bound in a genogroup- and strain-dependent manner to molluscan gastrointestinal carbohydrate structures (HBGA-like moieties and sialic acid-residues) [60]. As known “hotspots” for the accumulation of multiple norovirus strains [212,213], bivalve molluscs have been postulated to present opportunities for infectious HuNoV inter-and intragenotype co-infection (thus facilitating subsequent viral recombination within the host), and have been pinpointed as high-risk vectors for the introduction of novel recombinant strains into the human population [182,214]. In a similar context, bivalve molluscs, as potential interfaces of shared species exposure through filtration of human and animal waste, have also tentatively been implicated as a putative way of introducing both human and different animal noroviruses into a single host [182,215]. Norovirus outbreaks are often reported in the context of communal dining at restaurants, festivals, picnics, schools, cruise ships and military bases [216,217,218,219] or in institutional settings such as hospitals and care homes, where spread of infection from a common-source exposure is facilitated by enclosed living quarters and reduced personal hygiene [146,220,221,222]. In the context of PPE donning and doffing (this particularly in the context of the ongoing coronavirus pandemic), contamination of healthcare personnel, but particularly untrained users, with tenacious oral or respiratory pathogens that may contaminate masks or respirators has become a concern [196,197].

Recently, wild birds and rodents were named as new potential HuNoV transmission routes; GI and GII HuNoV genome copies were detected in faecal samples of gulls and crows (31%) and rats (2%), albeit with high ct values, implicating them as possible mechanical carriers, capable of spreading HuNoVs in the environment and possibly transmitting the virus to humans directly or indirectly by contaminating foods [223]. Determination of the replication capability of HuNoVs in these new tentative carriers (e.g., by detection of viral antibodies in blood or whole virus particles in faeces) is still pending. The high ct values suggest a possible mismatch between GI/II primers and probes, suggesting that the detected viruses might more feasibly be gull- and crow-specific avian noroviruses.

### 7.4. Seasonality of Human Norovirus Infections

Human norovirus infections follow a typical seasonality with incidents peaking during the winter months from October to March in the northern hemisphere [224]. While not fully elucidated, this pattern is attributed to a complex combination of host, climactic environmental, and viral factors. On the host side, peaks in norovirus infections are linked to changes in societal behaviour, an upsurge in hospitalisations due to other infectious diseases, and fading herd immunity. Inverse linear associations of norovirus laboratory reports and daily temperatures have been reported, linking cold, dry conditions to higher norovirus activity. Norovirus levels typically peak in northern winters in sewage [225,226], freshwater [227,228], and seawater as conditions for norovirus persistence in waters are improved by colder water temperatures and reduced solar irradiation [224,225,229].

### 7.5. Reservoirs

The excretion of infectious norovirus from persistently infected individuals [114,189] is purported to be one of the sources of norovirus outbreaks. Not only has the involvement of chronic shedders in hospital outbreaks indicated them to be a reservoir for nosocomial transmission of noroviruses [146], but persistently infected patients have also been suggested to contribute to HuNoV transmission as reservoirs for emerging strains. Intra-host evolution via point mutation accumulation [230,231] and the acquisition of superinfections over the protracted period of persistent infections [143] implicate persistently infected patient cohorts as potential reservoirs for novel HuNoV variants [213]. Multiple phylogenetic analyses have identified viral populations in persistently infected patients to be highly diverse and genetically distinct from viruses circulating in the general population [147,232]. The within-host viral variation via the acquisition of point mutations in chronic shedders is typically not random but has been shown to be a result of positive selection, as evidenced both by nonsynonymous versus synonymous substitution ratios (>1) and the clustering of amino acid changes at VP1 blocking epitopes (hypervariable P2 domain) and HBGA binding sites on the capsid surface [139,230,231,233,234]. Indeed, the intra-host emergence of antigenically distinct strains comparable to the variation between chronologically predominant GII.4 strains has been observed, suggesting that in certain individuals the evolution during a persistent norovirus infection translates into relevant phenotypic variability, thus, potentially selecting for viruses able to escape herd immunity to earlier isolates [235]. At an average of five to nine mutations per 100 days [231] or 1.85 to 2.66 × 10^−2^ substitutions per nucleotide site per year (s/n/y) in the viral capsid gene [234], norovirus evolution rates in immunocompromised hosts are generally significantly elevated as compared with those in healthy hosts. The process, whereby norovirus strains can acquire enough mutations to constitute novel epidemic subtypes within weeks to months (on a global scale this would normally take years), has been attributed to the particularities of a reduced but constant intra-individual selection pressure in immunocompromised hosts [139,231,236]. Siebenga et al. reported that the number of VP1 amino acid changes selected per time in intra-individual quasispecies was higher in patients with intermediate immunocompromise than in severely immunocompromised patients [139]. Over the prolonged period of persistent norovirus infections, superinfections with a second genotype have been shown to occur in a sixth of patients; in such cases, temporary mixed infections can be detected in a single sample [143]. Mixtures of norovirus strains further heighten the complexity of intra-individual quasispecies in immunocompromised hosts and provide opportunities for viral recombination, constituting another possible factor towards driving the emergence of new epidemic strains [182,237].

While multiple analyses have highlighted the diversity of norovirus variants in immunocompromised patients and have shown that chronic variants have the propensity to rapidly generate novel variants, the contribution of this diversity to norovirus evolution at the inter-host population level is still unclear. Recent mathematical modelling based on the standard epidemiological categorisation of susceptible, infected, and recovered individuals, suggested that despite the capacity of immunocompromised hosts to generate significant diversity, the relative isolation and rarity of such hosts limits their impact on broader pathogen evolution and epidemiology. Specifically, only a minor role for immunocompromised individuals in shaping large scale evolutionary patterns and processes and the global emergence of new HuNoVs was inferred [238].

While the reservoir of novel norovirus strains is yet to be definitively identified, norovirus diversity could also be originated at inter- and intra-host levels in otherwise healthy populations of different age groups (from infants in day care centres [239] to adults in the context of communal living and dining, as described above). Thus, mutations could arise during transmission events which present an evolutionary bottleneck in outbreak settings, and/or during shedding in healthy individuals [232,237].

The lack of certitude regarding the source of newly emerging HuNoVs and the close genetic relatedness between certain animal and human norovirus have generated interest in the possible role of animals as a potential zoonotic reservoir for emerging strains [66]. More than two thirds of human emerging infectious diseases are thought to originate from animal reservoirs [240]; for other members of the *Caliciviridae* family, interspecies transmission has been reported [241,242]. The, as yet, unproven existence of a zoonotic potential for noroviruses has long been discussed, the potential interfaces of shared species exposure being food, water, or animal contact. Despite known noroviruses exhibiting marked host specificity, the discussion about interspecies and/or zoonotic transmission is fuelled by the close relationship of certain animal and human norovirus strains, detection of HuNoVs in animal faeces, detection of antibodies against HuNoVs in swine, and the demonstration of experimental HuNoV GII infection in gnotobiotic pigs [15,220,243,244]. Questions concerning species barrier determinants preventing HuNoV infection of murine cells were recently resolved with the identification of a CD300lf proteinaceous receptor as the primary determinant of MNV species tropism. All other components of cellular machinery required for norovirus replication are conserved between humans and mice [43]; expression of MNV CD300 family receptor molecules rendered non-murine mammalian cells susceptible to MNV infection [245]. If the key to cross-species transmission lies only at a structural virus-host receptor level, this presents ORF1/2 norovirus recombination (discussed in [182]), by which a nascent recombinant virus gains a complete novel capsid protein set, in an interesting light, in that a “lucky” intragenogroup recombination event between two co-infecting viruses might tender a zoonotic/interspecies recombinant. Indeed, putative GIV.2_GVI.1interspecies recombinant FNoVM49, detected in a cat captured near a Japanese oyster farm in 2015 [215], may have originated via a similar mechanism. However, since conclusive data supporting inter-species transmission is yet lacking, the continuous emergence of new HuNoV through zoonotic events is unlikely.

## 8. Detection and Typing of Noroviruses

### 8.1. Diagnostic Methods

Since norovirus infections present a major public health issue, rapid diagnosis is vital for the initiation of appropriate control measures to curtail viral spread and curb the extent of outbreaks. According to the typical clinical presentation of norovirus infections, the Kaplan criteria can assist in diagnosis when laboratory resources are unavailable to determine an outbreak aetiology. Developed from pooled data of gastroenteritis outbreaks between 1967 and 1980, the Kaplan criteria consist of four patterns that characterise norovirus outbreaks. Accordingly, stool cultures negative for bacterial pathogens, mean (or median) duration of illness of 12–60 h, vomiting in greater than or equal to 50% of cases, and a mean (or median) incubation period of 24–48 h satisfy the criteria for a Norwalk-like infection [115]. While a useful diagnostic aid in discriminating confirmed foodborne gastroenteritis outbreaks due to noroviruses from those due to bacteria with a reportedly high specificity (99%), these criteria are only moderately sensitive (68%) [246], necessitating further laboratory confirmation of the viral aetiology.

Electron microscopy, utilised for the first ever identification of norovirus particles in stool [5], permits rapid and direct visualisation of noroviruses, however, lacks sensitivity and facile implementation (highly trained personnel is a prerequisite to its use), rendering it ineligible for routine diagnostics [9]. In lieu of this costly method, and in the absence of a stable and inexpensive HuNoV cell culture system, routine laboratory diagnostics for noroviruses are typically either performed via immunological assays or amplification of viral nucleic acids.

While the development of a broadly reactive norovirus antigen enzyme immunoassay (EIA) has proven challenging owing to the number of antigenically distinct HuNoV genotypes and the continuous antigenic drift of certain strains [247], several EIAs are commercially available for the detection of GI and GII antigens in stool specimens. Most commercial kits consist of solid-phase, sandwich-type immunoassays and include combinations of multiple cross-reactive monoclonal and polyclonal antibodies. Sensitivity and specificity of these kits, typically around 70% and 90%, respectively, are subject to significant variation depending on the viral load and norovirus genotypes present in the sample. The clinical context of sample collection (sporadic case versus outbreak) and the number of samples tested are recognised to influence the sensitivity of EIAs to such an extent that their use, while undoubted for rapid screening of multiple faecal samples during an outbreak of acute gastroenteritis, is not recommended in interpreting test results from sporadic cases and that negative results should be further confirmed by molecular methods (RT-PCR) [248,249]. Similarly, immunochromatographic lateral flow assays, designed for rapid and uncomplicated testing of individual faecal samples, have been shown to have a varying, genogroup-dependent sensitivity and, while useful for preliminary screening in outbreaks, their negative results should be verified by RT-PCR [250].

Amplification-based techniques for the detection of noroviruses in clinical samples, environmental samples, and food and water include conventional RT-PCR [251] and one- or two-step quantitative real-time RT-PCRs (RT-qPCR) [252]. Most contemporary assays use genogroup-specific oligonucleotide primers and fluorescent probes typically targeting a small conserved genome region at the ORF1/ORF2 junction [253]. Increasingly, such assays are multiplexed, allowing simultaneous detection of multiple norovirus genotypes within different genogroups, for example, the simultaneous detection of GI and GII strains [254,255] or GI, GII, and GIV strains [256]; several different multiplex molecular gastrointestinal diagnostic pathogen platforms are commercially available [257].

Quantitative RT qPCR assays, which implement either intercalating dyes [258], or fluorescent probe-based chemistries [256], can be used to determine the amount of nucleic acid in a sample. However, a distinction between infectious and non-infectious virus particles is not possible and virus detection by RT-qPCR does not necessarily correlate with a true infectious norovirus burden. Methods to evaluate the correlation between genomic copies and infective norovirus particles are under investigation. Amongst these, the binding long-range PCR [259] has been proposed to assess genome integrity and both the use of a ligand binding step prior to RT-qPCR [260,261] or viability PCR assays which include pretreatment with intercalating dyes are increasingly utilised to investigate capsid integrity [262,263]. Comparison of RT-qPCR results with newly developed HuNoV infectivity assays (further discussed below) may help determine cycle threshold cut-offs for clinical diagnostic RT-qPCRs, allowing estimation of infectious virus burdens to help guide infection control [264,265].

Increasingly, the spectrum of analytical techniques is being widened. Promising developments in the field include biosensors (such as monoclonal antibodies, aptamers, porcine gastric mucin, and HBGAs), investigated for their potential of concentrating noroviruses, microarray-based assays [266] and omics-based analyses [267,268].

### 8.2. Genotyping

With the increasing implementation of molecular methods in norovirus diagnostics, virus typing through (partial) sequence analysis has become increasingly common. The web-based, open access Norovirus Automated Genotyping Tool (Version 2.0, NoroNet, Bilthoven, The Netherlands) for sequence-based typing, available online from the NoroNet website of the Dutch National Institute for Public Health and the Environment (http://www.rivm.nl/mpf/norovirus/typingtool; accessed on 01.06.2021), provides direct and internationally standardised genotyping of noroviruses. On the basis of genetic homology and phylogenetic inferences, the tool assigns sequences to a norovirus genogroup, maps query sequences to a specific location on the reference genome(s) and offers information on RdRp- and capsid affiliation on either side of the ORF1/2 overlap. Briefly, the tool, updated periodically with new names and reference strains, employs a typing algorithm on ORF1 and ORF2 sequences of GI and II noroviruses, starting with BLAST analysis of the query sequence against a reference set of *Caliciviridae* sequences. This is followed by phylogenetic analysis of the query sequence and a subset of the reference sequences to assign norovirus genotype and/or variant (GII.4), with profile alignment, construction of phylogenetic trees, and bootstrap validation [269]. The Human Calicivirus Typing Tool (HuCaT) (https://norovirus.ng.philab.cdc.gov/bctyping.html; accessed on 01.06.2021) uses a set of curated reference sequences (identical to those of the NoroNet tool) that are compared to query sequences using a k-mer- (DNA substring) based algorithm and accurately assigns genotypes to queries [270]. For more detailed information of norovirus detection and typing, we refer to the 2020 survey of analytical techniques for noroviruses by Liu et al. [268] and the 2021 description of HuCaT by Tatusov et al. [270].

## 9. Immunity to Human Noroviruses

Many gaps remain in the understanding of natural immunity to HuNoVs. In addition to genetic resistance to infection based on secretor status (see above), with non-secretors representing as much as 20% of the European population [271], norovirus infection has been shown to result in development of clinical immunity. Immunity against HuNoV is postulated to include both cellular and humoral responses. Recent findings show that natural HuNoV infection results in the production of proinflammatory and anti-inflammatory cytokines [272], that HuNoV replication in zebrafish larvae results in a measurable innate response [273], and that the innate immune response partially restricts HuNoV replication in human intestinal epithelial cells (IECs) through interferon-induced transcriptional responses and production of proinflammatory and anti-inflammatory cytokines [274].

Humoral immunity to HuNoV has been considered to be stronger and more long lasting than cellular immunity. According to human challenge studies, first estimates of immunity duration suggested short-term, adaptive immunity to homotypic Norwalk re-challenge with high viral doses to last from two months to two years [275] or for longer than six months [55]. Epidemiological data and mathematical modelling have since suggested that naturally induced immunity in the absence of major strain changes may actually last for much longer and potentially span up to a decade [276]. While seroprevalence studies have shown an estimated 90% of adult populations to be seropositive to norovirus [277], probably only a small fraction of the total HuNoV specific antibodies represent partial or even absolute neutralising antibodies, i.e., correlates of protection that mediate reduced infection or disease severity [278]. Strain dependent differences in the induction of protective immune responses [279], antigenic diversity, and known lack of heterotypic cross-protection between certain norovirus genogroups, genotypes, and strains [280] further confound the determination of immunity duration [281].

In mice, the innate STAT-1-dependent immune response plays a key role in limiting MNV replication [16], however, the initial innate response alone is often insufficient to protect against acute MNV infection and humoral immunity has been shown to play a critical role in MNV clearance [282] and protection from subsequent infection [279].

## 10. Treatment and Prophylaxis

Despite the clinical significance and societal burden of norovirus infections, neither approved antivirals nor licensed vaccines are yet available.

### 10.1. Antivirals

While medical intervention is rarely needed in typical norovirus infections of immunocompetent individuals, safe and effective antivirals are essential for treatment of high-risk, persistently infected immunocompromised individuals, and other vulnerable populations (juvenile/elderly). In the absence of specific therapeutic measures, treatment is focused on providing supportive care such as rehydration and electrolyte replenishment.

Research efforts towards antiviral development have been furthered by a deeper understanding of the norovirus replicative cycle and recent breakthroughs in culturing HuNoVs. Direct acting antiviral therapies target various stages of the norovirus replication cycle [283,284]. Strategies to prevent norovirus attachment and entry include HBGA binding inhibition via various glycomimetic compounds [285,286,287] and passive immunotherapy with monoclonal antibodies [288] or nanobodies [289]. The activity of NS6 protease inhibitors depends on preventing polyprotein processing by the viral NS6. Candidate drugs targeting this step include broad-spectrum antivirals that covalently bind to the catalytic site of 3C or 3C-like proteases [290], enzymatic transition state inhibitors, or analogues [291]. Compounds targeting viral polymerase NS7 to interfere with norovirus replication comprise chain-terminating and mutagenic nucleoside analogues as well as non-nucleoside inhibitors. Nucleoside analogues under investigation include the cytidine analogue 2′-C-methylcytidine [292] and its derivatives, and purine analogues favipiravir [293] and ribavirin, the latter of which is licensed to treat chronic hepatitis C infections [145,294,295]. Modified C-nucleosides have been recently identified as viable starting scaffolds for further optimisation of nucleoside-based inhibitors of norovirus replication [296]. Their inhibitory effects are attributed to multiple modes of action including chain termination, provocation of an error catastrophe scenario for the viral quasispecies via ambiguous base pairing (lethal mutagenesis), direct RdRp inhibition, and unbalancing of intracellular NTP pools [297,298]. Non-nucleoside inhibitors target binding pockets of the RdRp, thus, preventing conformational changes required for formation of an active replication complex [299]. Host factor drugs with the potential to treat norovirus infections include immunomodulators (type I, II, and III interferons) [300,301,302] and small molecule inhibitors that downregulate viral RNA secondary structure-binding host factors [284]. The thiazolide nitazoxanide, tested with varying outcomes in preliminary clinical trials against chronic HuNoV infections [303,304], may activate natural host antiviral defences but may also act through the direct inhibition of virus protein production, maturation, and/or assembly. A comprehensive review of progress made in the development of antivirals (this between 2010 and 2018) was provided in 2018 by Netzler et al. [283].

### 10.2. Vaccines

The development of HuNoV vaccines is desired to protect vulnerable populations (immunocompromised/juvenile/elderly) and high-risk groups, including health care workers, military personnel, and (cruise ship) travellers experiencing crowding conditions. Prophylactic applications may also include the vaccination of food handlers to reduce the occurrence of foodborne outbreaks.

Key challenges for norovirus vaccine development pertain to vaccine effectiveness in the face of norovirus strain diversity and continuing evolution, which call for multivalent vaccines and periodic updates to protect against a range of current and emerging epidemiologically important genotypes. Furthermore, the lack of a universally accepted correlate of protection against norovirus, documented varying seroresponse and uncertainty regarding the duration of long-term immunity conferred by norovirus infection or vaccination are barriers faced in norovirus vaccine development [305]. Nevertheless, a bivalent GI.1/GII.4 VLP vaccine [306] and a recombinant adenovirus vector vaccine expressing GI.1 or GII.4 VP1 with monovalent or bivalent dosing [307], are currently in clinical trials. Further vaccines have been approved for clinical trial testing or are in the preclinical phase of development [281,308]. Three recent reviews discuss challenges to and advances in HuNoV vaccine development [281,305,308].

## 11. Norovirus Evolution

The evolution of noroviruses is shaped both by the accumulation of point mutations (genetic drift) and viral recombination (genetic shift), and the interplay of these two pivotal evolutionary processes.

### 11.1. Human Norovirus Mutation Rates and Sources of Point Mutation

The norovirus RdRp, sharing functional and structural features with other RNA virus polymerases, is the primary determinant of norovirus genetic diversity [309]. In vitro RdRp fidelity assays have demonstrated overall HuNoV mutation rates to lie within the range of those typically described for RNA viruses, but pinpointed strain-dependent differences [310]. Globally predominant GII.4 strains had five- to 36-fold higher mutation rates (average of 7.95 × 10^−4^ substitutions per nucleotide site or 5.97 ± 1.96 substitutions per genome replication event) as compared with less frequently detected strains, GII.P21 (1.53 × 10^−4^ or 1.15 substitutions per genome replication event) and GII.7 (2.21 × 10^−5^ or 0.17 substitutions per genome replication event).

Recently, single-cycle viral replication of a Norwalk virus infectious cDNA clone transfected into human embryonic kidney cells yielded a mutation rate estimate of 1.5 × 10^−4^ s/n/c [311]. Interestingly, a large fraction of spontaneous mutations constituted U-to-C and A-to-G substitutions occurring as bouts of mutations in the same RNA molecule. Such sequence changes are characteristic of adenosine to inosine editing (inosines subsequently base-pair with cytosines) by double-strand RNA-dependent adenosine deaminases (ADARs) [312], suggesting that host-driven extrinsic norovirus hypermutation acting on double-stranded replication intermediates may be a source of norovirus diversity comparable to intrinsic viral RdRp fidelity. In depth analysis of spontaneous mutations in clinical GII.4 samples has supported the hypothesis that hypermutation may reflect a relevant mutational process in noroviruses [311].

### 11.2. Evolutionary Rates of Human Noroviruses

Early bioinformatics analysis of published ORF2 sequence data revealed strain dependent differences in norovirus evolutionary rates, estimating 1.7-fold higher average rates of evolution within GII.4 capsid sequences (3.9 × 10^−3^ nucleotide substitutions per site per year (n/s/y)) than other noroviruses (GII.3, GII.3[Pb], GII.7 with 1.9 × 10^−3^, 2.4 × 10^−3^, and 2.3 × 10^−3^ n/s/y, respectively) [310]. Higher ratios of nonsynonymous to synonymous amino acid changes in GII.4 capsids were held to indicate that GII.4 strains experience faster rates of antigenic drift than other norovirus strains as a probable consequence of their higher RdRp mutation rates [310]. Nonsynonymous mutations for norovirus GII.4 and all other analysed genotypes (albeit at lower numbers) were shown to cluster to common structural surface-exposed residues of the hypervariable P2 capsid domain, corresponding to known HBGA-binding targets and hypervariable GII.4 “evolution hotspots” [313], suggesting that these sites are likely to be subject to immune-driven selection [310].

Other long-term evolutionary analyses of archival norovirus sequences have calculated similar population-level evolutionary rates for GII.4 VP1 capsid sequences (4.3 × 10^−3^ n/s/y) and have identified preferential sites for evolution under positive selection to be located in the VP1 shell domain as well as P2 [314,315]. However, in contrast to previous observations, evolutionary rates of various non-GII.4 genotypes, for example, GII.3 VP1 (4.16 × 10^−3^ n/s/y) [181], GII.2[P2] (1.75 × 10^−3^ n/s/y), and GII.2[P16] (2.37 × 10^−3^ n/s/y) [316] have been estimated to be close to those of GII.4 strains. Differences in mutation rates may provide higher diversity at a given time (e.g., after a recombination event) and so confer an advantage to GII.4 strains; however, they seem to have a limited impact on overall norovirus evolutionary rates. Strain-dependent differences of norovirus evolutionary patterns are, thus, not entirely attributable to differences in viral RdRp fidelity and remain to be fully elucidated.

Full-genome deep sequencing analyses have revealed that evolutionary rates are not uniform across the norovirus genome, with surface- and immune-exposed regions experiencing more variation than less malleable sections. Correspondingly, ORF2 (VP1) and ORF3 (VP2)-specific rates are typically higher than those reported for ORF1 (NS). Within ORF1, regions encoding NS1/2 and NS4 have been shown to exhibit the highest levels of change [317,318]. Overall norovirus evolutionary dynamics at inter-host population levels may differ from intra-host dynamics where, subsequent to transmission typically characterised by a strong genetic bottleneck, evolutionary rates fluctuate by several orders of magnitude dependent on the host immune status [231,232,236] (as described in chapter 1.6.6).

### 11.3. Impact of Human Norovirus Diversification via Point Mutation Accumulation

The emergence of GII.4 variants is commonly ascribed to the accumulation of novel VP1 GII.4 amino acid mutations (linear evolution with intermediate periods of stasis), while non-GII.4 genotypes experience limited changes and can persist for decades with minimal VP1 modification as so-called static genotypes [181,237,315,319].

The emergence of both GII.4 and non-GII.4 viruses has been linked to changes in the viral RdRp, highlighting it, and potentially other nonstructural proteins, as drivers of norovirus evolution. Thus, the emergence of certain GII.4 variants (since their establishment as prevalent genotype in the mid-1990s) has been associated with mutations in the GII.4 RdRp gene [320] or acquisition of a new viral polymerase via recombination (the genetic diversity of GII.4 variants due to recombination is discussed in chapter 2.3) [237,321]. Both the predominance of re-emerging (2016–2017) [316,322] recombinant GII.2 [P16] viruses [316,319] and GII.17[P17] viruses between 2013 and 2015 [323] have been putatively associated to substitutions in the viral RdRp. Notably, single HuNoV RdRp point mutations have been experimentally demonstrated to affect replication kinetics [310].

Thus, norovirus diversification and emergence are associated (in varying measure) with changes to two regions of the norovirus genome, nonstructural protein-encoding ORF1 and VP1-encoding ORF2.

### 11.4. Murine Norovirus Evolution via Point Mutation Accumulation

In vitro mutation rates have been inferred for representative genome regions of MNV isolate MNV1-CW1 [324]. Mutation rates were shown to not significantly differ between regions encompassing partial coding sequences for NS1/2, NS5, NS6, and NS7 within ORF1, where they were within the same range as those reported for various HuNoV strains, but were estimated to be at least one order of magnitude higher for partial ORF2, -3, and -4 sequences [324]. Interestingly, the existence of defective RNAs or defective interfering particles, commonly associated with the population dynamics of error-prone virus replication [325], was indicated by diverging infectious norovirus virus titres and genomic copy values determined during MNV serial passaging [324].

Highlighting the importance of point mutation as an evolutionary mechanism for noroviruses, a single point mutation in NS1/2 (changing aspartic acid to glutamic acid) has been shown to dramatically alter the biological behaviour of a MNV, rendering non-persistent MNV1-CW3 persistent and causing an increased growth of CW3 in the proximal colon, a tissue reservoir of MNV persistence [91,165]. Furthermore, a single amino acid substitution in VP1 has been shown to be sufficient for MNV attenuation (or the reversion thereof) in vivo [326]. Other in vivo assays have shown that single point mutations modulating MNV RdRp fidelity may affect MNV pathogenesis; Arias et al. demonstrated a high-fidelity MNV-3 NS7 active-site mutant to exhibit delayed replication in vivo (but not in vitro) and reduced transmission between hosts, suggesting that the generation of sufficient genetic diversity (via a low-fidelity RdRp) may be linked to efficient intra-host virus transmission [327]. Conversely, artificially increased mutagenesis above the inherently high mutation rates of noroviruses has been shown to lead to extinction of MNV populations [293], highlighting the norovirus RdRp as an important target for the development of anti-noroviral therapies.

### 11.5. Norovirus Recombination

Norovirus recombination can create chimeric viruses to generate new recombinants and further contribute to norovirus strain diversification by combining and modifying existing mutational profiles. Intragenotypic recombination has long been postulated to be a driving force of GII.4 noroviruses. Increasingly, emergence and re-emergence of different intragenotype recombinants have been reported [182,328,329]. The subject of norovirus recombination was recently reviewed by our group; we refer to our 2018 review [182] for further information.

## 12. Model Systems to Study Norovirus Biology

### 12.1. In Vivo Model Systems for Human Noroviruses

Early volunteer challenge studies and epidemiological observations of HuNoVs in their natural hosts have yielded important in vivo data to further the understanding of HuNoV infections [55,134,271]. However, the interpretation of results from such studies may be complicated by small sample sizes, variations in susceptibility to infection, previous history of exposure, and cross-reactivity of antibodies. In addition, volunteer studies may pose potential health risks to participants. Thus, a robust HuNoV animal model has long been sought.

Various non-human primates have been tested as HuNoV infection models [330]. While neither baboons, common marmosets, cotton top tamarins, nor cynomolgus seem susceptible to HuNoV infection [331], rhesus macaques and chimpanzees produce serum antibodies and shed virus upon oral HuNoV infection but do not develop clinical symptoms [331,332,333]; only infection of pigtail macaques has been shown to result in typical clinical illness including vomiting, thus, potentially presenting a model to study the emetic response to HuNoVs [334].

Large animal models for symptomatic HuNoV infection include gnotobiotic pigs and calves. Infection of gnotobiotic piglets with a GII.4 HuNoV results in mild diarrhoea, faecal shedding of viral RNA, expression of viral RNA in intestinal enterocytes and extra-intestinal lymphoid tissues, and seroconversion [335,336]. Prolonged HuNoV infections and viral dissemination beyond the intestine have been observed in gnotobiotic pigs with a severe combined immunodeficiency phenotype [337]. Gnotobiotic piglets provide a useful experimental model as the pig intestine anatomy resembles that of humans and protection from disease provides a valuable readout in vaccine trials and testing of therapeutics [338,339]. Gnotobiotic calves infected orally with HuNoV develop diarrhoea associated with intestinal damage and faecal viral shedding for up to six days, as well as local and systemic immune responses [340].

Double knockout recombination activation gene (Rag^-/-^) and common gamma chain (γc^-/-^) deficient BALB/c mice support subclinical HuNoV GII.4 replication upon infection via the intraperitoneal route [341]. The model has been used to assess the anti-HuNoV activities of antiviral compounds [342]. However, since these mice cannot be infected orally and lack both gut-associated lymphoid tissues and the ability to produce numerous cytokines and mature B and T cells, the model cannot recapitulate typical HuNoV infection.

Recently, multiple HuNoV GI and GII strains were shown to replicate to high titres in cells of both the hematopoietic lineage and the intestine of zebrafish larvae (*Danio rerio*) following yolk inoculation (larval food reserve) [273]. Yielding over three orders of magnitude (3log_10_) increases in GII.4 viral RNA copies, zebrafish larvae were shown to constitute a simple and robust in vivo HuNoV replication model and were also demonstrated to be suited to antiviral studies.

### 12.2. Human Norovirus Tropism and In Vitro Culture of Human Noroviruses

In lieu of a stable HuNoV culture system, HuNoV in vitro assays were, until very recently, conducted using the Norwalk virus replicon [102] and/or virus-like particles (VLPs). The Norwalk virus RNA replicon consists of an intact ORF1 and ORF3, and an ORF2 disrupted by a neomycin gene engineered into the VP1-encoding region (thus blocking expression of intact VP1). Self-replicating and stably expressed following transfection into cell lines of human (Huh-7) or hamster (BHK21) origin, the replicon has proven useful for the study of HuNoV genome replication and screening of antiviral compounds [295,343]. The RNA replicon is complemented by VLP systems, in which expression of capsid protein VP1 results in the self-assembly of recombinant VLPs that are morphologically and antigenically indistinguishable from native HuNoV virions and consequently represent useful tools to study physical virion properties, antibody responses, and attachment factor interactions [39].

Notwithstanding the utility of these two systems, the fact that the understanding of HuNoV biology has lagged behind that of other positive strand RNA viruses has been, in great part, due to the difficulties historically associated with robust in vitro HuNoV propagation [344,345]. In turn, issues with HuNoV cell culture stem from the uncertainties still surrounding HuNoV tropism and the lack of a known (proteinaceous) entry receptor (see above). Recent data support a dual cell tropism of epithelial cells and nonepithelial cells of hematopoietic origin both in vivo [346] and in vitro [347] and illustrate a complex interplay with the host microbiome [79,348].

Currently, two different HuNoV cell culture systems successfully capitalise on this dual tropism. The development of the in vitro BJAB human B cell line demonstrated that HuNoV (and MNV) can either infect B cells directly or in a coculture system in which the virus must cross a confluent epithelial monolayer to access underlying B cells. Productive GII.4 HuNoV infection of B cells required the presence of the HGBA-expressing commensal bacteria (or free synthetic HBGA), identifying them as a stimulatory cofactor for bridging norovirus attachment to and infection of B cells [108,348]. This and other available data directed the development of a working model for norovirus intestinal infection, whereby noroviruses bind to specific glycans expressed on the surface of members of the gut microbiota and/or enterocytes, and then are transcytosed across the polarised intestinal epithelial barrier to gain access to their target immune cells [109,349]. Notably, this model provides an explanation for how noroviruses may achieve co-infection of host cells in conditions when the number of cells far outweighs that of virions; multiple genetically distinct virions can be effectively concentrated by binding to the surface of a single bacterium, thereby increasing the opportunity for co-infection [350,351]. While the technical simplicity and use of a commonly used cell line are strengths of the BJAB assay, current drawbacks are the modest level of viral replication and varying reproducibility.

In a technically more complicated approach, but with more robust infection levels overall, cultivation of multiple HuNoV strains has recently been demonstrated in stem cell derived, human intestinal enteroid (HIE) cultures (epithelial mini guts) which recapitulate the multicellular, physiologically active human intestinal epithelium [73,352]. Grown from single multipotent stem cells of the human intestinal crypts (isolated from endoscopic biopsies), HIEs can be maintained continuously as three-dimensional cultures. Differentiation into distinct mature cell types present in the epithelium, such as absorptive enterocytes, multiple secretory cells (Paneth cells, goblet cells, enteroendocrine cells, and tuft cells), and the M cells of Peyer’s patches can be achieved by modifying culture conditions [353,354]. Propagation (and limited passaging) of HuNoVs in enterocytes of differentiated HIEs (seeded into monolayers) has been shown to be dependent on HBGA expression in a strain-dependent manner (secretor-negative HIEs are permissive to GII.3, but not GII.4 replication). Bile acids have been shown to assist productive infection of certain strains (GI.1, GII.3, and GII.17) [47], but bile is not necessary for cultivation of HuNoV GII.4/Sydney and may also be dispensed with when specific media are used [355]. The expense and complexity of the HIE system, relatively low sensitivity of the cultures to infection, issues with sustained passaging, and the unresolved basis for strain specific replication requirements remain challenges faced in the ongoing enhancement of HuNoV HIE cultures [352].

Different in vivo and in vitro HuNoV assays have all provided invaluable tools to dissect the norovirus life cycle. However, there is still a lack of detailed understanding of norovirus replication and significant questions remain unanswered due to the technical limitations of many of these experimental systems. Experimental models of HuNoV infection are discussed in detail in the 2019 review by Todd and Tripp [330].

### 12.3. The Murine Norovirus—An In Vivo and In Vitro Human Norovirus Surrogate

The genetically and biologically closely related murine norovirus (MNV) combines the advantages of easy in vivo infection of a cost-effective, genetically tractable, bona-fide native host [16], efficient and robust in vitro culture systems [167,356], and availability of tools for genetic manipulation (including both DNA- and RNA-based reverse genetics systems) [163,357,358]. Caveats to the model include differences between HuNoV and MNV carbohydrate attachment factors and proteinaceous receptors (see above), the fact that HuNoVs replicate in intestinal enterocytes, a cellular tropism that MNV does not seem to share, and the typically asymptomatic nature of MNV infections in wild-type mice. In vivo MNV infections of adult immunocompetent and immunocompromised mice as well as those of neonatal mice are described above. The adult in vivo models have long yielded valuable information concerning the biology of a norovirus in its natural host [167]. The newly described model of norovirus diarrhoea in which key clinical features of HuNoV disease are mirrored in MNV-infected neonatal mice will open up new avenues of research and the finding that disease severity is regulated by viral genetics (MNV-3 and MNV-CR6 cause a reduced incidence of diarrhoea relative to MNV-1) will facilitate identification of viral virulence determinants [169].

Until very recently, MNVs were the only cultivable noroviruses, replicating efficiently and to high titres in cultured bone marrow-derived murine macrophages (RAW264.7 cells) [167,356] and murine-derived microglial cells (BV-2 cells) [359] as well as B cells (M12 and WEHI-231), where peak titres are reached one day later than in RAW264.7 macrophages [348].

## 13. Conclusions

The fact that the understanding of HuNoV biology lags behind that of other positive strand RNA viruses, is, in great part, due to the difficulties historically associated with robust in vitro HuNoV propagation. In recent years, novel model systems have provided new opportunities for the study of HuNoVs and previous knowledge gaps are progressively being filled. Recent advances in the field comprise an updated classification system [10], the description of alternative virus-like protein (VLP) morphologies [48], and the further elucidation of the functions of both structural and nonstructural viral proteins and their roles in the norovirus replicative cycle [36,52,92,101]. Important milestones include new insights into cell tropism [40,44], host- and microbial attachment factors and receptors [43,59,60,61,71,72,73,79,360], interactions with the cellular translational apparatus [80,83,84], and viral egress from cells [110]. Noroviruses have been detected in previously unrecognised host species [18,19]; detection itself is facilitated by the novel analytical techniques that increasingly supplement established molecular methods [259,261,262,263,265]. New potential HuNoV transmission routes and/or viral reservoirs have been postulated [223,230,233]. Recent in vivo and in vitro findings have added to the understanding of host immunity in response to norovirus infection [272,273,274]. While neither approved vaccines nor small molecule treatments are yet available, vaccine development has progressed to preclinical and even clinical trial testing of candidates [281,306,307,308] and ongoing development of therapeutic agents includes promising direct-acting small molecules and host-factor drugs [145,292,296,302]. Many of these discoveries have been facilitated by novel in vitro and in vivo culture systems that have inspired fascinating new avenues of norovirus research.

## Data Availability

Not applicable.
